# 577. Week 48 Results of a Phase 2 Study Evaluating Once-weekly Oral Islatravir Plus Lenacapavir

**DOI:** 10.1093/ofid/ofae631.015

**Published:** 2025-01-29

**Authors:** Amy E Colson, Gordon E Crofoot, Peter J Ruane, Moti N Ramgopal, Alexandra W Dretler, Ronald G Nahass, Gary I Sinclair, Mezgebe Berhe, Fadi Shihadeh, Shan-Yu Liu, Stephanie Klopfer, Sharline Madera, Hadas Dvory-Sobol, Martin Rhee, Elizabeth G Rhee, Jared Baeten, Joseph J Eron

**Affiliations:** Community Resource Initiative, Boston, MA; The Crofoot Research Center, Houston, Texas; Ruane Clinical Research, Los Angeles, California; Midway Immunology & Research Center, Fort Pierce, Florida; Metro Infectious Disease Consultants, Decatur, Georgia; ID Care, Hillsborough, New Jersey; Prism Health North Texas, Dallas, Texas; North Texas Infectious Diseases Consultants, Dallas, Texas; Gilead Sciences, Foster City, California; Gilead Sciences Inc, Foster City, California; Merck & Co., Inc., Rahway, New Jersey; Gilead Sciences, Inc., Foster City, California; Gilead Sciences, Foster City, California; Gilead Sciences, Foster City, California; Merck & Co., Inc., Rahway, New Jersey; Gilead Sciences, Foster City, California; University of North Carolina at Chapel Hill School of Medicine, Chapel Hill, North Carolina

## Abstract

**Background:**

Both islatravir (ISL), a nucleotide reverse transcriptase translocation inhibitor, and lenacapavir (LEN), a capsid inhibitor, have potent anti-HIV-1 activity and pharmacokinetic profiles permitting once-weekly oral dosing. Week (W) 24 data (primary endpoint) from the current Phase 2 study were previously reported (CROI 2024); weekly oral ISL 2 mg + LEN 300 mg maintained high rates of viral suppression (HIV-1 RNA < 50 copies/mL) with no clinically relevant decreases in CD4+ T-cells or lymphocytes, which had been previously observed with higher ISL doses. Here, we report W48 results.

**Table:**
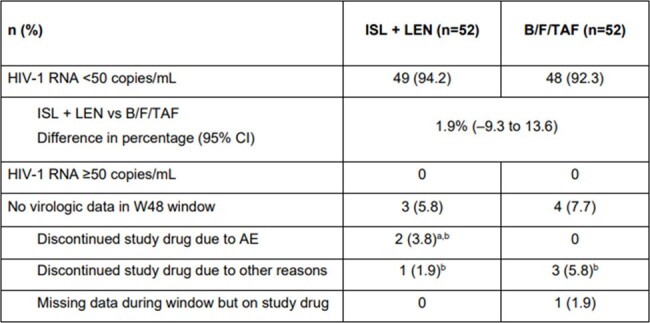
Week 48 Virologic Outcome Data by FDA Snapshot Algorithm ^a^AEs leading to study discontinuation included large intestine perforation and renal colic (n=1); and hepatitis B (n=1). ^b^Last available on-treatment HIV-1 RNA <50 copies/mL. AE, adverse event; B/F/TAF, bictegravir/emtricitabine/tenofovir alafenamide; FDA, Food and Drug Administration; ISL, islatravir; LEN, lenacapavir; W, week.

**Methods:**

In this Phase 2, randomized, open-label, active-controlled study (NCT05052996), virologically suppressed adults on bictegravir/emtricitabine/tenofovir alafenamide (B/F/TAF) were randomized 1:1 to receive weekly oral ISL 2 mg + LEN 300 mg or to continue daily B/F/TAF. Virologic outcomes (using FDA-defined snapshot algorithm), adverse events (AEs), CD4+ T-cells, and lymphocytes were assessed.

**Results:**

Overall, 104 participants were randomized and dosed; median age (range) was 40 (26–76) years, and 19 (18.3%) were assigned female at birth. At W48, 49/52 (94.2%) participants in the ISL + LEN group had HIV-1 RNA < 50 copies/mL vs 48/52 (92.3%) participants in the B/F/TAF group (**Table**). No participants had HIV-1 RNA ≥50 copies/mL at W48. The most common AEs in ISL + LEN participants (≥10%) were upper respiratory tract infection (n=7; 13.5%), COVID-19 (n=6; 11.5%), and diarrhea (n=6; 11.5%). No Grade ≥3 AEs, serious AEs, or AEs leading to study drug discontinuation were related to study drug. Two (3.8%) participants discontinued ISL + LEN due to AEs unrelated to study drug (both reported at W24: large intestine perforation and renal colic [n=1]; hepatitis B [n=1]). At W48, there were no significant differences between ISL + LEN vs B/F/TAF groups in mean change from baseline in CD4+ T-cells (–12 vs –29/µL; P=0.88) or lymphocytes (–0.07 vs –0.03 x 10^3^/µL; P=0.23).

**Conclusion:**

Oral weekly ISL + LEN maintained high rates of viral suppression at W48 and was well tolerated. There were no statistically significant between-group differences in CD4+ T-cell or lymphocyte changes. ISL + LEN has the potential to become the first weekly oral complete regimen for the treatment of HIV-1 infection.

**Disclosures:**

**Amy E. Colson**, Gilead Sciences, Inc.: Advisor/Consultant|ViiV: Honoraria **Gordon E. Crofoot, n/a**, AbbVie: Grant/Research Support|Gilead Sciences, Inc.: Grant/Research Support|Janssen: Grant/Research Support|Merck: Grant/Research Support|ViiV: Grant/Research Support **Peter J. Ruane, n/a**, Gilead Sciences, Inc.: Advisor/Consultant|Gilead Sciences, Inc.: Honoraria|ViiV: Advisor/Consultant|ViiV: Honoraria **Moti N. Ramgopal, n/a**, AbbVie: Honoraria|Gilead Sciences, Inc: Advisor/Consultant|Gilead Sciences, Inc: Honoraria|Merck: Advisor/Consultant|ViiV: Advisor/Consultant|ViiV: Honoraria **Alexandra W. Dretler, n/a**, AbbVie: Grant/Research Support|Gilead Sciences, Inc.: Grant/Research Support|ViiV: Advisor/Consultant|ViiV: Grant/Research Support **Ronald G. Nahass, MD**, Abbvie: Honoraria|Arbutus: Grant/Research Support|Gilead Sciences, Inc.: Grant/Research Support|Merck: Grant/Research Support|Vir: Grant/Research Support **Gary I. Sinclair, n/a**, Abbvie: Advisor/Consultant|Abbvie: Grant/Research Support|Abbvie: Honoraria|Gilead Sciences, Inc.: Advisor/Consultant|Gilead Sciences, Inc.: Grant/Research Support|Janssen: Advisor/Consultant|Janssen: Grant/Research Support|Janssen: Honoraria|Merck: Advisor/Consultant|Merck: Grant/Research Support|Merck: Honoraria|Theratechnologies: Advisor/Consultant|Theratechnologies: Grant/Research Support|Theratechnologies: Honoraria|ViiV: Advisor/Consultant|ViiV: Grant/Research Support|ViiV: Honoraria **Fadi Shihadeh, n/a**, Gilead Sciences, Inc: employee and shareholder **Shan-Yu Liu, PhD**, Gilead Sciences, Inc.: employee and shareholder **Stephanie Klopfer, n/a**, Merck & Co., Inc.: subsidiary and shareholder|Merck Sharp & Dohme LLC: employee **Sharline Madera, MD, PhD**, Gilead Sciences, Inc.: employee and shareholder **Hadas Dvory-Sobol, PhD**, Gilead Sciences, Inc.: employee and shareholder **Martin Rhee, MD**, Gilead Sciences, Inc.: employee and shareholder **Elizabeth G. Rhee, n/a**, Merck & Co., Inc.: subsidiary and shareholder|Merck Sharp & Dohme LLC: employee **Jared Baeten, MD, PhD**, Gilead Sciences, Inc.: employee and shareholder **Joseph J. Eron, MD**, Gilead Sciences: Advisor/Consultant|Gilead Sciences: Grant/Research Support|Invivyd: Safety Monitoring Board|Merck & Co: Advisor/Consultant

